# Clinical correlates of dopamine transporter availability in cross-sectional and longitudinal studies with [^18^F]FE-PE2I PET: independent validation with new insights

**DOI:** 10.1093/braincomms/fcae345

**Published:** 2024-10-02

**Authors:** Praveen Honhar, Faranak Ebrahimian Sadabad, Sule Tinaz, Jean-Dominique Gallezot, Mark Dias, Mika Naganawa, Yanghong Yang, Shannan Henry, Ansel T Hillmer, Hong Gao, Soheila Najafzadeh, Robert Comley, Nabeel Nabulsi, Yiyun Huang, Sjoerd J Finnema, Richard E Carson, David Matuskey

**Affiliations:** Department of Biomedical Engineering, Yale School of Engineering and Applied Sciences, Yale University, New Haven, CT 06511, USA; Radiology and Biomedical Imaging, Yale School of Medicine, New Haven, CT 06520, USA; Radiology and Biomedical Imaging, Yale School of Medicine, New Haven, CT 06520, USA; Department of Neurology, Yale School of Medicine, New Haven, CT 06510, USA; Radiology and Biomedical Imaging, Yale School of Medicine, New Haven, CT 06520, USA; Radiology and Biomedical Imaging, Yale School of Medicine, New Haven, CT 06520, USA; Radiology and Biomedical Imaging, Yale School of Medicine, New Haven, CT 06520, USA; Radiology and Biomedical Imaging, Yale School of Medicine, New Haven, CT 06520, USA; Radiology and Biomedical Imaging, Yale School of Medicine, New Haven, CT 06520, USA; Department of Biomedical Engineering, Yale School of Engineering and Applied Sciences, Yale University, New Haven, CT 06511, USA; Radiology and Biomedical Imaging, Yale School of Medicine, New Haven, CT 06520, USA; Department of Psychiatry, Yale School of Medicine, New Haven, CT 06511, USA; Radiology and Biomedical Imaging, Yale School of Medicine, New Haven, CT 06520, USA; Radiology and Biomedical Imaging, Yale School of Medicine, New Haven, CT 06520, USA; AbbVie, North Chicago, IL 60064, USA; Radiology and Biomedical Imaging, Yale School of Medicine, New Haven, CT 06520, USA; Radiology and Biomedical Imaging, Yale School of Medicine, New Haven, CT 06520, USA; AbbVie, North Chicago, IL 60064, USA; Department of Biomedical Engineering, Yale School of Engineering and Applied Sciences, Yale University, New Haven, CT 06511, USA; Radiology and Biomedical Imaging, Yale School of Medicine, New Haven, CT 06520, USA; Radiology and Biomedical Imaging, Yale School of Medicine, New Haven, CT 06520, USA; Department of Neurology, Yale School of Medicine, New Haven, CT 06510, USA; Department of Psychiatry, Yale School of Medicine, New Haven, CT 06511, USA

**Keywords:** [^18^F]FE-PE2I PET, DAT PET, Parkinson’s disease, DAT correlates

## Abstract

[^18^F]FE-PE2I PET is a promising alternative to single positron emission computed tomography–based dopamine transporter (DAT) imaging in Parkinson’s disease. While the excellent discriminative power of [^18^F]FE-PE2I PET has been established, so far only one study has reported meaningful associations between motor severity scores and DAT availability. In this study, we use high-resolution (∼3 mm isotropic) PET to provide an independent validation for the clinical correlates of [^18^F]FE-PE2I imaging in separate cross-sectional (28 participants with Parkinson’s disease, Hoehn–Yahr: 2 and 14 healthy individuals) and longitudinal (initial results from 6 participants with Parkinson’s disease with 2-year follow-up) cohorts. In the cross-sectional cohort, DAT availability in the putamen and substantia nigra of patients with Parkinson’s disease showed a significant negative association with total motor severity (*r* = −0.59, *P* = 0.002 for putamen; *r* = −0.46, *P* = 0.018 for substantia nigra), but not tremor severity. To our knowledge, this is the first observed association between motor severity in Parkinson’s disease and DAT availability in the substantia nigra. The associations with motor severity in most nigrostriatal regions improved if tremor scores were excluded from motor scores. Further, we found significant asymmetry in DAT availability in the putamen (∼28% lower DAT availability within the more-affected side of the putamen), and DAT-based asymmetry index for the putamen was correlated with asymmetry in motor severity (*r* = −0.60, *P* = 0.001). In the longitudinal study, [^18^F]FE-PE2I PET detected significant annual percentage reduction of DAT availability at the individual level in the putamen (9.7 ± 2.6%), caudate (10.5 ± 3.8%) and ventral striatum (5.5 ± 2.7%), but not the substantia nigra. Longitudinal per cent reduction in DAT availability within the putamen was strongly associated with increase in motor severity (*r* = 0.91, *P* = 0.011) at follow-up, demonstrating the high sensitivity of [^18^F]FE-PE2I PET in tracking longitudinal changes. These results provide further evidence for the utility of [^18^F]FE-PE2I as an important *in vivo* PET biomarker in future clinical trials of Parkinson’s disease.

## Graphical Abstract

**Graphical Abstract:**
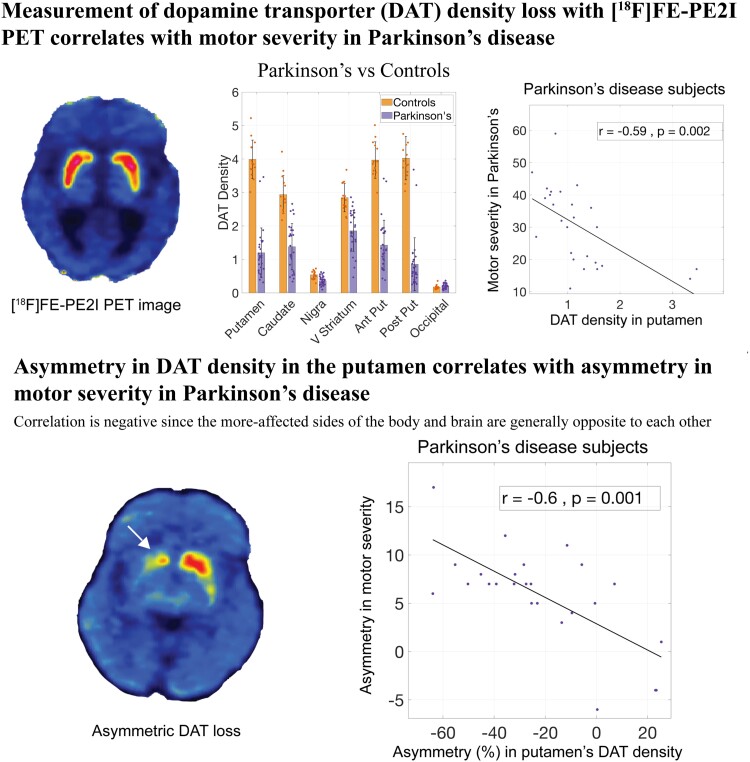

## Teaser

Honhar et al. examined correlates of dopamine transporter loss in Parkinson's disease using [^18^F]FE-PE2I PET imaging. Dopamine transporter loss in all nigrostriatal regions (including nigra) was significantly correlated with motor impairment, and the asymmetry in dopamine reduction in the putamen was correlated with the extent of asymmetry in motor symptoms.

## Introduction

Parkinson’s disease is the fastest-growing, neurodegenerative disorder that significantly affects the nigrostriatal dopaminergic system of the brain.^1^ The pathophysiology of Parkinson’s disease is characterized by the accumulation of misfolded oligomers and aggregates of α-synuclein (Lewy bodies or neurites), primarily not only in the substantia nigra but also in the nigrostriatal tract and striatum.^2^ Dopamine transporter (DAT) is a protein that is expressed on the cell bodies, axons and pre-synaptic terminals of dopaminergic neurons within the nigrostriatal pathway.^3^ For decades now, single positron emission computed tomography (SPECT)–based molecular imaging of DAT using [^123^I]FP-CIT has been used for differential diagnosis of clinical Parkinsonism from other motor disorders, such as essential tremor.^4,5^ In the last decade, research efforts have focused on PET imaging of DAT, which offers higher resolution and sensitivity than SPECT.^6-11^

[^18^F]FE-PE2I is a promising PET radiotracer to image DAT within the nigrostriatal system. The longer half-life of this tracer, compared with [^11^C]PE2I, makes this a commercially viable imaging agent for clinical use. In addition, [^18^F]FE-PE2I not only has high affinity (*K*_D_ ∼ 12 nM)^12^ and selectivity for DAT^13^ but also has faster kinetics.^14^ Several clinical studies have validated the use of reference region–based kinetic models [such as simplified reference tissue model (SRTM) or Logan reference model] without the need for arterial blood sampling.^8,14,15^ Similarly, outcomes for simplified quantification (standardized uptake value ratio) that either use an early or late pre-defined time-window,^9^ and methods that can correct the bias in standardized uptake value ratio have been developed,^16^ making [^18^F]FE-PE2I PET suitable for translation to wider clinical practice.

The improved quantification offered by PET imaging of DAT has already provided tangible benefits to clinical research. Better quantification of DAT loss in the substantia nigra can help diagnose scans without evidence of dopamine deficit in the striatum.^6^ Due to its small test–retest variability,^9^ [^18^F]FE-PE2I PET can be also used to track progressive dopaminergic deficits within an individual.^9,11^ Recent work compared effect sizes for [^18^F]FE-PE2I PET with SPECT-based DAT imaging and estimated that a treatment trial would likely need two to three times lower sample size (compared with SPECT-based DAT imaging) for a 30–50% treatment effect.^11^ These studies establish [^18^F]FE-PE2I PET as a promising biomarker in Parkinson’s disease. However, another desired aspect of a biomarker is its association with clinical measures of disease severity and also its ability to track longitudinal changes in the clinical measures. Despite evidence for the dopaminergic losses in the substantia nigra and dorsal striatum being responsible for motor impairment in Parkinson’s disease,^17,18^ several previous studies with [^18^F]FE-PE2I PET in smaller cohorts failed to report significant associations between DAT loss and clinical motor scores.^6,7,9,11^ However, a recent cross-sectional study, which employed a larger cohort and consistent medication state between imaging and clinical examination did indeed report a significant association between DAT loss in putamen (and sensorimotor striatum) and clinical motor scores.^10^

Despite these promising results and favourable head-to-head comparison against [^123^I]FP-CIT,^7^ [^18^F]FE-PE2I PET imaging has not been approved by Food and Drug Administration as an imaging procedure to evaluate Parkinson’s disease and other Parkinsonian syndromes. To our best knowledge, clinical studies with [^18^F]FE-PE2I PET in Parkinson’s disease have not been performed in patient cohorts from the USA/North America. Therefore, the main objective of this work was to evaluate the clinical significance of [^18^F]FE-PE2I PET imaging in an independent North American cohort. Towards this goal, we present findings from an ongoing, prospective clinical study, with separate cross-sectional [Parkinson’s disease (*n* = 28) and controls (*n* = 14)] data and initial results from a longitudinal [6 Parkinson’s disease subjects, mean follow-up period: 2.2 ± 0.7 years) study. In addition to estimating effect sizes for group differences in the cross-sectional study and annual percentage reduction (APR) of DAT in longitudinal data, we also collected clinical motor scores in the medication ‘OFF’ state, so that a better validation of its association with nigrostriatal DAT availability could be performed. We hypothesized the existence of a significant negative association between the clinical motor burden of the disease and DAT availability in the substantia nigra and putamen. Finally, we studied hemispherical asymmetry in DAT availability (with respect to the symptom onset side) and its association with the asymmetry in the clinical manifestation of motor symptoms due to Parkinson’s disease.

## Materials and methods

### Study participants: demographic information

Twenty-eight individuals with Parkinson’s disease (mean ± SD age: 64.4 ± 6.7 years, 14 women, 14 men, see Table 1 for detailed information) and 14 healthy individuals (mean ± SD age: 58.5 ± 5.3 years, 7 women, 7 men) underwent DAT-PET imaging after a bolus injection of [^18^F]FE-PE2I. Parkinson’s disease diagnosis was confirmed by a qualified movement disorders neurologist in accordance with Movement Disorders Society’s (MDS) guidelines.^19^ Additionally, six individuals with Parkinson’s disease had a follow-up DAT-PET scan at the 2-year time point (Table 2). All Parkinson’s disease subjects withheld their morning medications on the day of PET (last dose at least 12 h prior to PET).

**Table 1:** Clinical characteristics and radiotracer details of cross-sectional study

	Parkinson’s disease	Healthy controls	*P*-value
*n*	28	14	
Age	64.4 ± 6.7	58.5 ± 5.3	0.006
Sex (female:male)	14:14	7:7	
MDS-UPDRS III	30.4 ± 12.1		
Hoehn and Yahr	2.0 ± 0.0		
Disease duration (from symptom onset)	6.1 ± 4.5		
Injected dose (MBq)	149 ± 35	157 ± 27	0.46
Injected mass (ng/kg)	7.4 ± 5.2	11.6 ± 10.0	0.08

Reported values are mean ± SD.

**Table 2:** Clinical characteristics and radiotracer details of the longitudinal Parkinson’s cohort (*n* = 6)

	Visit 1	Visit 2	*P*-value
Age	64.3 ± 4.4 (57–69)	66.5 ± 4.1 (60–72)	0.40
Follow-up (in years)		2.2 ± 0.7 (1.2–3.2)	
Sex (female:male)	2:4	2:4	
MDS-UPDRS III	24 ± 12.3 (14–42)	29.5 ± 12.7 (18–50)	0.003^a^
Hoehn and Yahr	2.0 ± 0.0	2.0 ± 0.0	
Disease duration (from symptom onset)	4.1 ± 3.4 (1–10)	6.3 ± 4.1 (2–13)	0.34
Injected dose (MBq)	141 ± 43 (81–173)	165 ± 10 (152–178)	0.16^a^
Injected mass (ng/kg)	6.8 ± 2.1 (5.3–10.1)	12.8 ± 8.1 (4.5–20.7)	0.20^a^

Reported values are mean ± SD (range).

^a^ *P*-values from paired *t*-tests.

Study participants underwent comprehensive medical history, physical and neurological examination and routine blood tests as part of recruitment to this study. Exclusion criteria included significant medical or neurological illness (other than Parkinson’s disease), cognitive impairment, evidence of alcohol or substance abuse, pregnancy (verified using tests at screening and day of imaging), breast feeding and contraindications to MRI.

The study was conducted under protocols approved by the Yale University Human Investigation Committee and the Yale University Radiation Safety Committee. Written informed consent was obtained from all participants after complete explanation of procedures. All imaging studies reported here were performed at the Yale PET and magnetic resonance research centres between 2019 and 2023.

### Magnetic resonance imaging

All participants underwent a T_1_-weighted, magnetization prepared rapid acquisition gradient echo (echo time = 2.44 ms, inversion time = 900 ms, repetition time = 2500 ms, flip angle = 9^°^, voxel size = 1 mm^3^ isotropic) sequence MRI on a 3-Tesla Siemens Prisma scanner. All magnetic resonance images were visually inspected for structural abnormalities and were used for co-registration with PET images.

### DAT PET

All participants underwent a dynamic PET acquisition (up to 60 min post-injection) on the high-resolution research tomograph (HRRT, Siemens CTI, Knoxville, TN, USA) after intravenous injection with [^18^F]FE-PE2I. [^18^F]FE-PE2I was synthesized using previously published methods^20^ with an average yield of 3.4 ± 1.8% (non-decay corrected), high radiochemical purity (97.1 ± 1.7%) and high molar activity (240.4 ± 123.4 GBq/µmol, see Supplementary material for detailed information on radiosynthesis and quality control). [^18^F]FE-PE2I was administered as a bolus injection using an automated infusion pump (Harvard PHD 22/2000, Harvard Apparatus). Arterial sampling was not performed as previous studies have validated the use of binding potential (BP_ND_) computed through SRTM for quantification of this radiotracer in healthy controls and individuals with Parkinson’s disease.^8,15^

Head motion was monitored throughout the PET acquisition using an optical tracking system (Polaris Vicra, NDI Systems, Waterloo, Ontario, Canada). PET data were reconstructed using the previously described MOLAR algorithm^21^ with corrections for motion, attenuation (assessed through a 6-min transmission scan on the HRRT performed before radiotracer injection), scattered and random coincidences and detector dead time.

### Image analysis: PET outcomes and quantification

This study relied on two PET outcomes—BP_ND_ that reflects DAT availability and relative tracer uptake (*R*_1_) that may be interpreted as an index of relative cerebral blood flow. First, parametric images of BP_ND_ and *R*_1_ were generated using the SRTM2 fits (with population average *k*_2_′ for cerebellum = 0.096 min^−1^) to voxel time activity. Note that we validated SRTM2 against the gold-standard SRTM for this tracer (see Supplementary Figs 1, 2, 6 and 7), with excellent agreement in regional values between the two methods.

Regional values of BP_ND_ were then computed in key dopaminergic nigrostriatal regions (putamen, caudate, substantia nigra and ventral striatum). For *R*_1_, regional values were computed for the putamen, caudate and cortical lobes to investigate large-scale blood flow changes in Parkinson’s disease. These regions of interest (ROIs) were defined using the anatomical automatic labelling for statistical parametric mapping (SPM) 2 (AAL) atlas^22^ (except for the substantia nigra), and these ROIs were then applied to the subject’s parametric SRTM2 images using a combination of non-linear and linear transformations as described previously.^23^ Substantia nigra ROI was defined using an in-house template that was previously created from [^11^C]PHNO-PET imaging of healthy controls on the same HRRT scanner.^24,25^ All brain regions (except substantia nigra) were grey matter segmented using computational anatomy toolbox for SPM12 and corrected for partial volume effects using the Müller–Gartner algorithm,^26^ prior to computation of parametric images and regional PET outcomes.

### Quantification of clinical motor symptoms in the Parkinson’s cohort

Clinical rating of motor burden of the disease was assessed by a movement disorders neurologist using the MDS-Unified Parkinson’s Disease Rating Scale Part III (MDS-UPDRS Part III)^27^ in the medication off state. On average, MDS-UPDRS assessments were made 2.0 ± 2.1 months before DAT-PET imaging. In addition to the total motor scores, we calculated the scores for different motor phenotypes including tremor, postural instability/gait difficulty (PIGD) and akinetic/rigid.^28^ Sum of the items 3.10 (gait), 3.11 (freezing of gait) and 3.12 (postural stability) was defined as the PIGD score. Sum of the items 3.15–3.18 (rest, postural, and kinetic tremor amplitude and constancy of the limbs and lip/jaw) was defined as the tremor score. The sum of the rest of the items (3.1 speech, 3.2 facial expression, 3.3 rigidity, 3.4–3.8 limb bradykinesia, 3.9 arising from chair, 3.13 posture and 3.14 body bradykinesia) was defined as the bradykinesia/rigidity score. Note that we classified item 3.13 (posture) with bradykinesia/rigidity as stooped posture in Parkinson’s disease has been primarily attributed to a combination of muscle rigidity, dystonia of the axial muscles, myopathy and impaired proprioception,^29,30^ as opposed to PIGD whose origin is likely due to problems in the closed loop circuit that integrates cognitive and sensory feedback with basal ganglia and brainstem–spinal locomotor centres.^31^

### Quantification of asymmetry in motor symptoms in the Parkinson’s cohort

We computed a clinical asymmetry score for motor burden presentation as the difference between the clinical scores for the more-affected (Maff) and less-affected (Laff) sides of the body (Maff: symptom onset side; Laff: side opposite to symptom onset). This clinical asymmetry score was generated as the sum of the differences (Maff−Laff) for all questions on MDS-UPDRS Part III that rate the motor burden on left and right sides of the body separately (3.3, 3.4, 3.5, 3.6, 3.7, 3.8, 3.15, 3.16 and 3.17).

### Cross-sectional analysis

Group differences and estimates for effect size (Cohen’s *d*) were calculated for DAT availability (BP_ND_) in the putamen, caudate, substantia nigra and ventral striatum. For *R*_1_, the group comparisons were made in the putamen, caudate and the four cortical lobes. Based on previous studies,^6-8,32^ we hypothesized significantly lowered DAT availability in all nigrostriatal regions and lowered *R*_1_ in the occipital–parietal areas.

Next, associations between clinical scores and PET outcomes were investigated. Due to relatively early stage of disease in the Parkinson’s disease cohort, 20 of 28 subjects with Parkinson’s disease had an identical PIGD score of 1 (range: 0–3); therefore, this score was dropped from further analyses due to the lack of inter-subject variability. MDS-UPDRS Part III total scores, tremor and bradykinesia/rigidity sub-scores were included in the correlation analyses. For each clinical score, we hypothesized a significant negative association with the DAT availability in the putamen and substantia nigra.

Asymmetry in regional DAT availability and relative cerebral blood flow were assessed by fitting regional PET data to the following linear model:


(1) $$\begin{array}{c} L_{i}-R_{i}=X_{o,i}\;+\;\beta_{i}\;X_{1,i}\;\; \end{array}$$

where subscript *i* refers to different (*i*th) regions, *L* and *R* refer to the PET outcome (either BP_ND_ or *R*_1_) in the left and right brain hemispheres respectively, $X_{1,i}$ = 0 for controls, −1 for Parkinson’s subjects for whom motor symptom onset was on the left side of the body and 1 for Parkinson’s subjects with motor symptom onset on their right side. Thus, the intercept term ($X_{o,i})$ is an estimate of mean regional left–right asymmetry in the PET outcome for controls, while the slope ($\beta_{i})$ represents the degree (percentage/100) of DAT-PET asymmetry observed for region *i* in the Parkinson’s disease cohort. $\beta_{i}$ measures the degree of asymmetry in DAT availability (Maff−Laff) in the patient group after accounting for any asymmetry in controls due to biological or methodological reasons. Please note that in the context of brain regions, the hemisphere that is contralateral to symptom onset side is considered as the ‘more-affected’ side or Maff and vice-versa. Inference and significance testing for PET asymmetry in region *i* was performed using the estimated mean value and standard error in $\beta_{i}$. Finally, we also computed the more typical regional asymmetry index for PET outcomes:


(2) $$\begin{array}{c} \;\left(\mathrm{AI}\;=\;\frac{\mathrm{Maff}-\mathrm{Laff}}{0.5\;(\mathrm{Maff}+\mathrm{Laff})}\;\right)\; \end{array}$$

That was then correlated to MDS-UPDRS Part III total and clinical asymmetry scores (*n* = 26 for correlations as two subjects did not undergo MDS-UPDRS examination). We hypothesized a significant asymmetry in the putamen DAT availability (Maff < Laff), which would be associated with asymmetry in motor scores.

Each individual analysis type was corrected for multiple comparisons (due to the analysis performed on multiple ROIs) by controlling for false discovery rate (FDR) at 5% (see the ‘Statistical analysis’ section).

### Longitudinal analysis

Changes in DAT availability in the putamen, caudate, substantia nigra and ventral striatum were calculated as APR using the following formula:


$$\mathrm{APR}\;(\text{\%})\;=\;100\;\times \frac{BP_{\mathrm{ND}}\;\mathrm{at}\;\mathrm{baseline}\;\text{-}\;\;BP_{\mathrm{ND}}\;\mathrm{at}\;\mathrm{follow}\text{-}\mathrm{up}}{BP_{\mathrm{ND}}\;\mathrm{at}\;\mathrm{baseline}}\times \frac{1}{\mathrm{years}\;\mathrm{between}\;\mathrm{baseline}\;\mathrm{and}\;\mathrm{follow}\text{-}\mathrm{up}}\;$$

This measure was chosen to facilitate comparison with previous longitudinal studies with [^18^F]FE-PE2I PET.^9,11^

To assess whether changes in DAT availability track with clinical motor burden of the disease, we correlated the percentage reduction in DAT availability in nigrostriatal regions (100 × $\frac{BP_{\mathrm{ND}}\;\mathrm{at}\;\mathrm{baseline}\;\text{-}\;\;BP_{\mathrm{ND}}\;\mathrm{at}\;\mathrm{follow}\text{-}\mathrm{up}}{BP_{\mathrm{ND}}\;\mathrm{at}\;\mathrm{baseline}}$) with individual changes in MDS-UPDRS Part III scores (follow-up−baseline).

Finally, a *post hoc* analysis was conducted to assess whether the changes in the PET-based DAT asymmetry index was associated with the changes in the asymmetry of motor score at the individual level. This analysis was only performed for DAT asymmetry in the putamen, since the cross-sectional study identified a significant asymmetry in the putamen, which was correlated with clinical asymmetry scores (see the ‘Results: Cross-sectional analysis’ section).

Though these analyses were also corrected for multiple comparisons, where appropriate, we recommend caution in the interpretation of these results due to the small sample size of the longitudinal cohort (*n* = 6, with two time points per subject).

### Statistical analysis

Statistical analysis was performed in R (v 4.2.2) and MATLAB (v2022a, MathWorks, Natick, MA, USA). All PET outcome measures (BP_ND_, *R*_1_) were approximately normally distributed (Shapiro–Wilk tests, *P* > 0.05). MDS-UPDRS Part III scores—total and without tremor/PIGD—were normally distributed, but the tremor score showed a strong non-normal distribution (Shapiro–Wilk tests, *P* < 0.001). Therefore, Pearson’s correlation coefficient was used to correlate MDS-UPDRS Part III and bradykinesia/rigidity scores with PET measures, while Spearman’s rank correlation coefficient was used for the correlations involving tremor scores.

Each analysis (such as a group difference or correlation with a specified covariate) was corrected for multiple comparisons arising from the statistical test being applied simultaneously to multiple brain regions using Benjamini–Hochberg procedure^33^ to control FDR at 5%. In the Results section, we first report the effect size and *P*-values based on univariate testing (without multiple comparison correction) and then clearly note which of those results survived the FDR correction procedure and, therefore, should be considered statistically significant.

## Results

### Cross-sectional analysis

#### Lower DAT availability and relative cerebral blood flow in Parkinson’s disease

**Figure 1:**
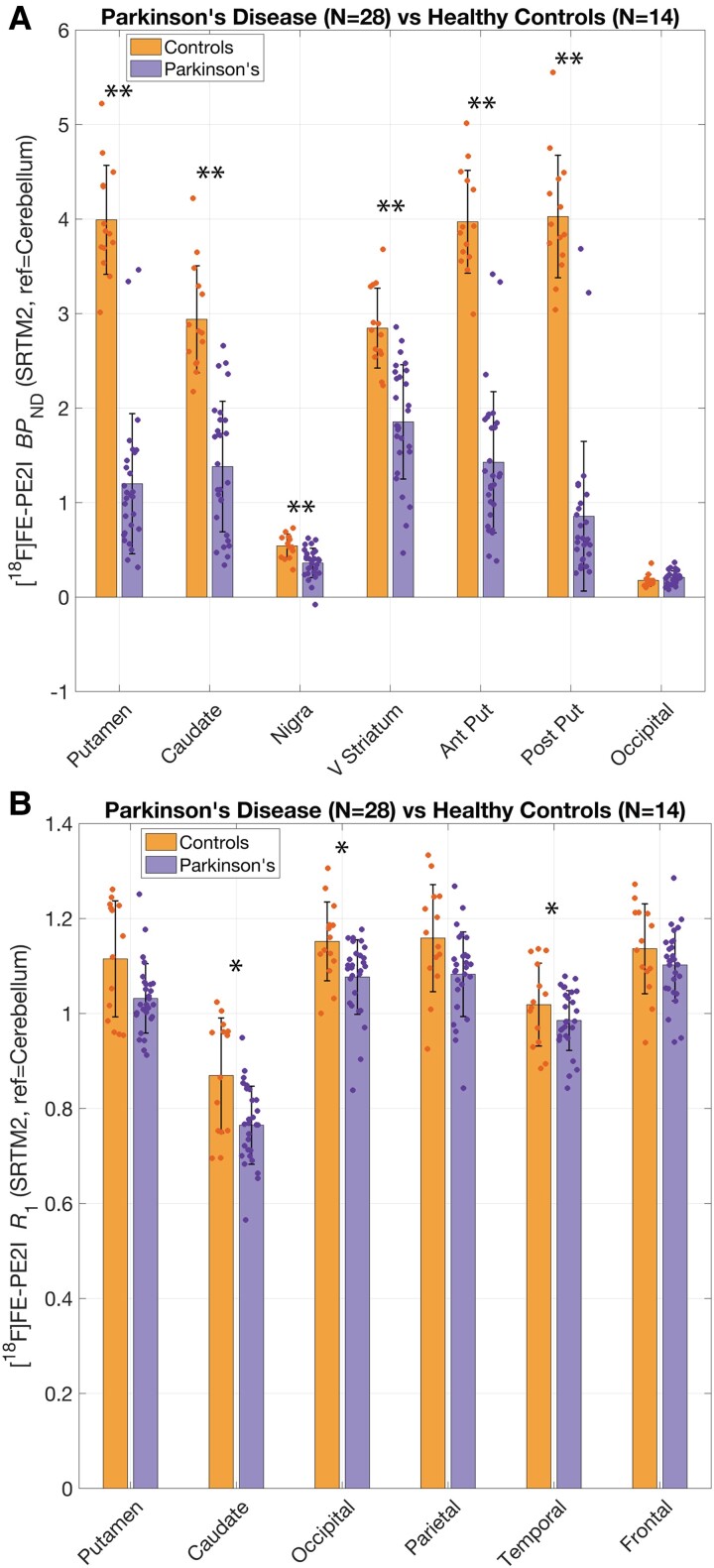
**Cross-sectional group comparison between Parkinson’s versus controls.** Group comparison of DAT availability (**A**) and relative cerebral blood flow (**B**) between Parkinson’s disease (*n* = 28) and healthy control (*n* = 14) subjects. Asterisks are shown only for differences that survived FDR correction: *P < 0.01, **P < 0.001 in univariate *t*-tests. DAT, dopamine transporter; BP_ND_, binding potential (relative to non-displaceable fraction); SRTM2, simplified reference tissue model 2; FDR, false discovery rate; ref, reference region; V Striatum, ventral striatum; Ant Put, anterior putamen; Post Put, posterior putamen.

The DAT BP_ND_ results are shown in Fig. 1A and summarized in Table 3. The Parkinson’s disease cohort, compared with controls, showed significantly lower DAT BP_ND_ in all nigrostriatal ROIs. All group differences in nigrostriatal ROIs were statistically significant after controlling for FDR. In addition, we performed two exploratory analyses—first, a simple division of the putamen into its anterior and posterior parts was performed by a coronal plane perpendicular to the mid-intercommissural (anterior commissure-posterior commissure) line.^34^ The posterior putamen was more affected by the disease as indicated by a 23% larger DAT loss on the posterior side compared with the anterior side. Finally, we also computed DAT BP_ND_ in the occipital lobe, which has been used as an alternate reference region in previous studies.^8^ Indeed, there were no significant differences in DAT availability in controls and patients in this region.

**Table 3:** BP_ND_ values across Parkinson’s disease and heathy controls (HC) groups

Region	Parkinson’s disease group (*n* = 28)	HC group (*n* = 14)	*P*-value	% Difference	Cohen’s *d* (magnitude)
Putamen	1.20 (0.74)	3.99 (0.58)	3.99 × 10^−15^	−69.9	4.03
Caudate	1.38 (0.69)	2.94 (0.57)	4.04 × 10^−9^	−53.0	2.39
Substantia nigra	0.36 (0.16)	0.54 (0.13)	0.0002	−33.3	1.23
Ventral striatum	1.85 (0.61)	2.85 (0.42)	2.13 × 10^−7^	−34.9	1.80
Anterior putamen	1.43 (0.75)	3.97 (0.54)	1.10 × 10^−14^	−64.1	3.70
Posterior putamen	0.86 (0.79)	4.03 (0.65)	3.49 × 10^−15^	−78.7	4.24
Occipital lobe	0.21 (0.07)	0.18 (0.06)	0.92	17.6	0.46

Values reported are mean (SD).

Figure 1B shows the regional [^18^F]FE-PE2I *R*_1_ values between Parkinson’s disease and control cohorts. Compared with controls, relative blood flow was lowered in Parkinson’s in the caudate (∼12%, *P* = 0.009, Cohen’s *d* = 1.10), putamen (∼7%, *P* = 0.03, Cohen’s *d* = 0.90), temporal (∼11%, *P* = 0.0024, Cohen’s *d* = 1.4) and occipital lobes (∼7%, *P* = 0.009, Cohen’s *d* = 0.94). Out of these, only the reductions in caudate, temporal and occipital lobes survived FDR correction. The data also showed an increased relative blood flow in the parietal lobe (∼9%, *P* = 0.03, Cohen’s *d* = 0.85); however, this did not survive FDR correction and was also the only example of a case where the non-partial volume–corrected results (∼7% decrease in parietal lobe, see Supplementary Fig. 3) did not support the partial volume–corrected results.

#### Association of DAT availability with clinical measures of motor performance and disease duration

**Figure 2:**
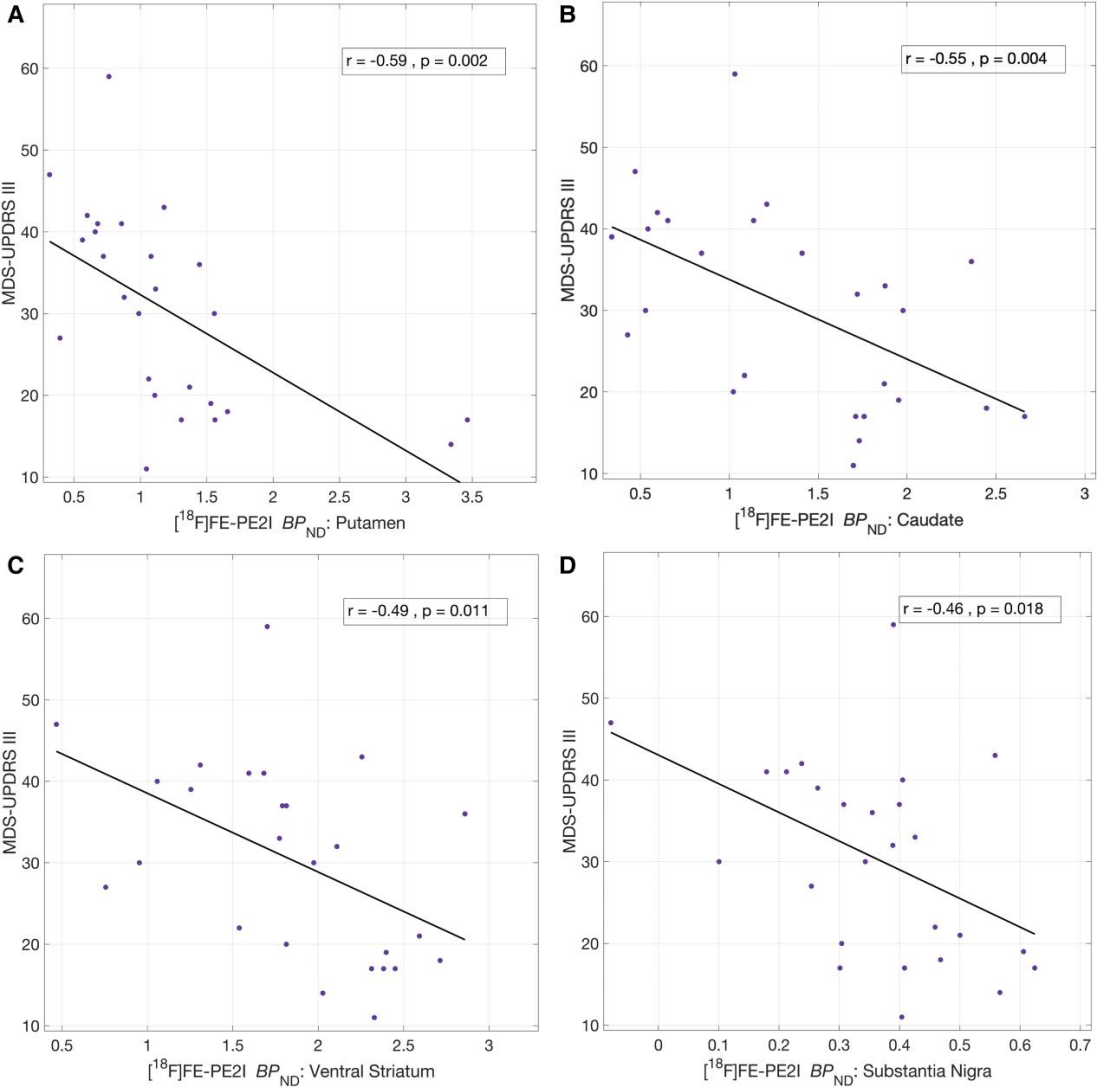
**Cross-sectional correlates of nigrostriatal DAT and MDS-UPDRS Part III scores in Parkinson’s disease.** Significant negative correlations (Pearson’s) between MDS-UPDRS Part III total scores and DAT BP_ND_ in the putamen (**A**), caudate (**B**), ventral striatum (**C**) and substantia nigra (**D**). All correlations survived FDR correction. Note that two subjects did not complete MDS-UPDRS examination, so *n* = 26 for these correlations. BP_ND_, binding potential (relative to non-displaceable fraction); MDS-UPDRS, Movement Disorders Society-Unified Parkinson’s Disease Rating Scale; FDR, false discovery rate; DAT, dopamine transporter.

Higher MDS-UPDRS part III scores indicate more severe motor disease. DAT BP_ND_ in the putamen (*r* = −0.59, *P* = 0.002), caudate (*r* = −0.55, *P* = 0.004), ventral striatum (*r* = −0.49, *P* = 0.011) and substantia nigra (*r* = −0.46, *P* = 0.018) showed significant negative associations with the total MDS-UPDRS Part III motor examination scores (Fig. 2). All correlations survived after controlling for FDR. The Part III scores also showed significant negative correlations with the DAT BP_ND_ in the anterior (*r* = −0.63, *P* = 0.001) and posterior (*r* = −0.50, *P* = 0.009) parts of the putamen (*post hoc* analysis).

**Figure 3:**
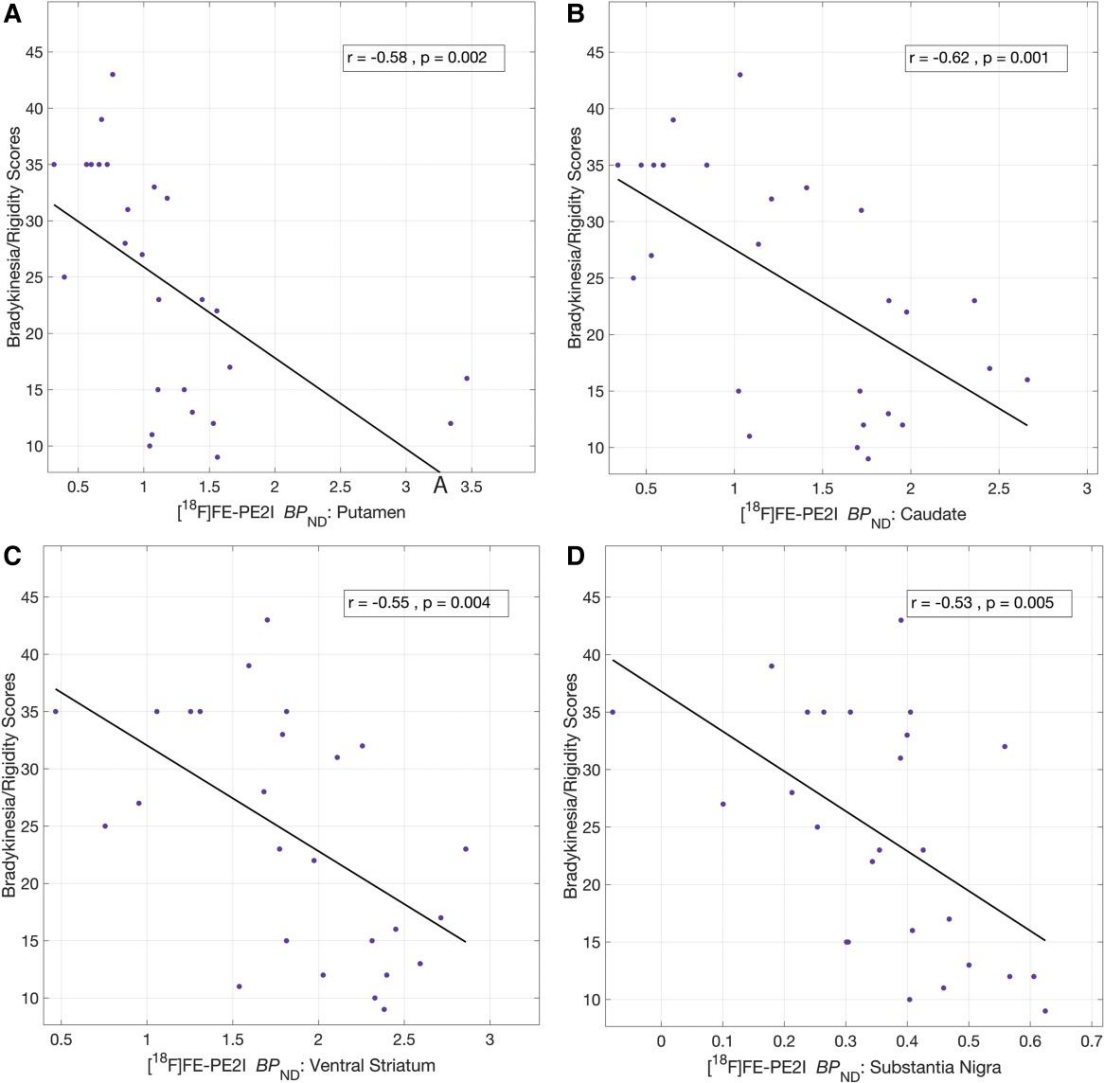
**Cross-sectional correlates of nigrostriatal DAT and bradykinesia/rigidity scores in Parkinson’s disease.** Significant negative correlations (Pearson’s) between bradykinesia/rigidity scores and DAT BP_ND_ in the putamen (**A**), caudate (**B**), ventral striatum (**C**) and substantia nigra (**D**). All correlations survived FDR correction. Two subjects did not complete MDS-UPDRS examination, so *n* = 26 for these correlations. BP_ND_, binding potential (relative to non-displaceable fraction); MDS-UPDRS, Movement Disorders Society-Unified Parkinson’s Disease Rating Scale; FDR, false discovery rate; DAT, dopamine transporter.

Next, associations between DAT availability in the nigrostriatal regions and tremor scores from clinical motor examination were investigated. No significant associations were found between DAT availability in any of the nigrostriatal regions and tremor scores. Upon removing tremor and PIGD from MDS-UPDRS Part III, the negative associations with DAT availability and the remaining bradykinesia/rigidity scores either remained unchanged (putamen: *r* = −0.58, *P* = 0.002) or slightly improved, as observed in the caudate (*r* = −0.62, *P* = 0.001), substantia nigra (*r* = −0.53, *P* = 0.005) and ventral striatum (*r* = −0.55, *P* = 0.004). All of these correlations remained significant after controlling for FDR (Fig. 3).

Disease duration (measured from self-reported symptom onset) was also negatively associated with DAT BP_ND_ in the ventral striatum (*r* = −0.50, *P* = 0.007), substantia nigra (*r* = −0.48, *P* = 0.010), putamen (*r* = −0.48, *P* = 0.011) and caudate (*r* = −0.43, *P* = 0.02, see Supplementary Fig. 4). These correlations remained significant after controlling for FDR.

No meaningful clinical associations were found between *R*_1_ and any measure of clinical motor severity or disease duration.

#### Asymmetry in DAT availability and its association with asymmetry in clinical motor symptoms

**Figure 4:**
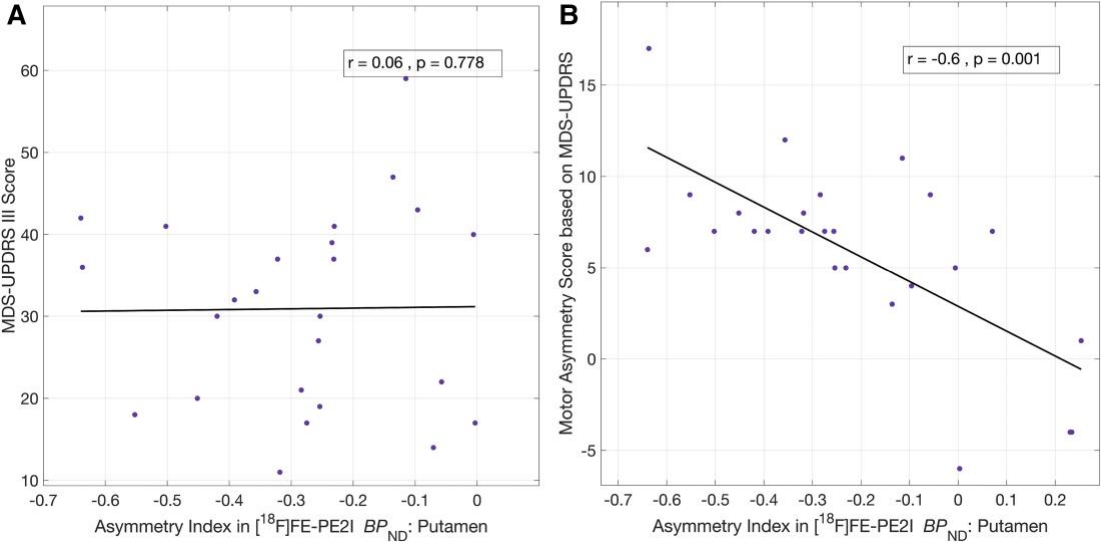
**Cross-sectional correlation between asymmetry in DAT-PET and motor severity in Parkinson’s disease.** Asymmetry of DAT availability in the putamen, $\frac{\mathrm{Maff}-\mathrm{Laff}}{0.5\;\;(\mathrm{Maff}+\mathrm{Laff})}$, was not associated with total MDS-UPDRS Part III scores (**A**) but was significantly correlated (Spearman’s) with the asymmetry in motor score (Maff-Laff) based on MDS-UPDRS Part III (**B**). Two subjects did not complete MDS-UPDRS examination, and one subject did not have separate motor scores for left and right sides of the body, so *n* = 25 for these correlations. DAT, dopamine transporter; Maff, more-affected side (symptom onset side for motor scores, contralateral to symptom onset side for brain regions); Laff, less-affected side (contralateral to symptom onset side for motor scores, ipsilateral to symptom onset side for brain regions); BP_ND_, binding potential (relative to non-displaceable fraction); MDS-UPDRS, Movement Disorders Society-Unified Parkinson’s Disease Rating Scale.

Using the linear model [Equation (1)], we observed significant asymmetry in regional DAT BP_ND_ in the putamen (∼28% lower BP_ND_ on the Maff side, *P* = 2.6 × 10^−9^) and caudate (∼18% lower BP_ND_ on the Maff side, *P* = 1.9 × 10^−5^), but not in the ventral striatum or substantia nigra. The asymmetry in the putamen and caudate survived FDR correction. Further, both the anterior and posterior parts of the putamen showed a similar asymmetry of ∼25–28%. While there was no association between asymmetry index of DAT BP_ND_ [Equation (2)] in the putamen and the total MDS-UPDRS Part III scores (Fig. 4A), a significant association was observed between the DAT BP_ND_ asymmetry score in the putamen and the total motor asymmetry score (*r* = −0.6, *P* = 0.001, Fig. 4B, survived FDR correction). Asymmetry index of caudate was not significantly associated with any clinical measure.

No asymmetry in relative cerebral blood flow (*R*_1_) was observed for any brain region.

### Longitudinal analysis

#### DAT binding potential (relative to non-displaceable fraction) in the striatum was significantly reduced at follow-up

The mean APR in DAT BP_ND_ was significant in the putamen, caudate and ventral striatum (Table 4). These results remained significant after controlling for FDR. The mean APR in the substantia nigra was not statistically significant (7%, *P* = 0.20). As exploratory analyses, we also computed the APR for the Maff and Laff putamen. The APR in DAT BP_ND_ was higher in the Laff putamen (10%), compared with Maff putamen (8.8%), at this stage of the disease. Individual trajectories for regional BP_ND_ values at baseline and follow-up are shown in Supplementary Fig. 5. No significant longitudinal change was observed for *R*_1_ values within the striatum and cortical lobes.

**Table 4:** APR in DAT availability for the longitudinal study

Region	Baseline BP_ND_	Follow-up BP_ND_	APR (%)	*P*-value (for APR)
Putamen	1.44 ± 1.00	1.17 ± 0.92	9.7 ± 2.6	0.0002
Caudate	1.31 ± 0.56	1.04 ± 0.55	10.5 ± 3.8	0.001
Substantia nigra	0.34 ± 0.11	0.30 ± 0.05	7.0 ± 11.6	0.20
Ventral striatum	1.87 ± 0.33	1.65 ± 0.41	5.5 ± 2.7	0.004
Maff putamen	1.29 ± 1.11	1.06 ± 0.96	8.8 ± 3.2	0.001
Laff putamen	1.59 ± 0.90	1.29 ± 0.88	10.0 ± 3.0	0.0004

Values reported are mean ± SD.

#### Change in DAT binding potential (relative to non-displaceable fraction) within putamen was associated with change in motor scores

**Figure 5:**
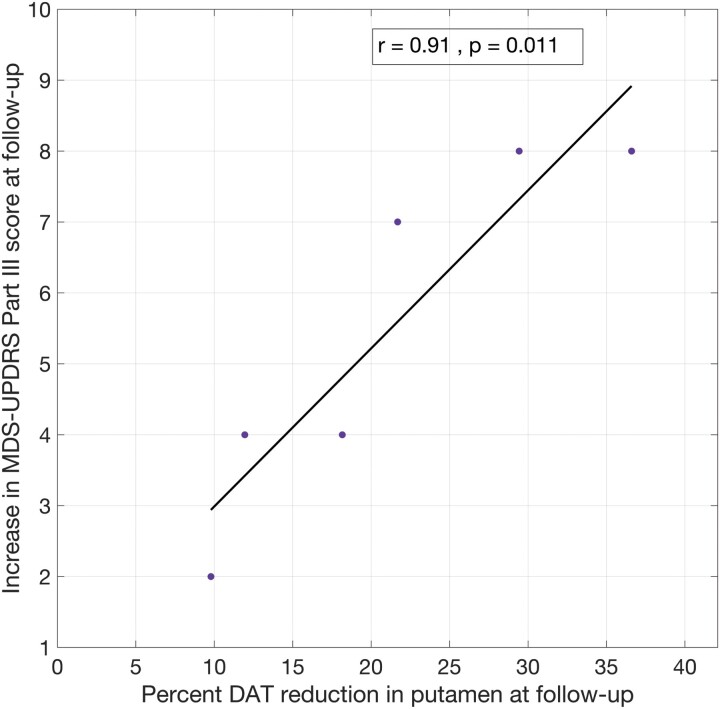
**Correlation between longitudinal change in DAT-PET and MDS-UPDRS Part III scores.** A significant association (Pearson’s, *n* = 6) was observed between percentage DAT reduction in putamen and increase in MDS-UPDRS Part III scores at follow-up (∼2 years after baseline). DAT, dopamine transporter; MDS-UPDRS, Movement Disorders Society-Unified Parkinson’s Disease Rating Scale.

There was an increase of 5.5 ± 2.5 points in MDS-UPDRS Part III scores in the Parkinson’s disease patients at follow-up. Similarly, there was a per cent decrease of 21.3 ± 10.3 in DAT availability in the putamen at follow-up. Within the four nigrostriatal regions, only per cent reduction in DAT BP_ND_ in the putamen was strongly associated with changes in MDS-UPDRS Part III scores (Pearson’s *r* = 0.91, *P* = 0.01, Fig. 5). This association remained significant after controlling for FDR. At this stage of the disease, change in MDS-UPDRS Part III scores was more strongly correlated with changes in DAT BP_ND_ in the Maff putamen (Pearson’s *r* = 0.96, *P* = 0.002), compared with the Laff putamen (Pearson’s *r* = 0.83, *P* = 0.04).

#### Change in DAT asymmetry within putamen was not associated with change in motor asymmetry score

There was a change of 0.5 ± 2.1 points in the motor asymmetry score in the patients with Parkinson’s disease at follow-up. The positive sign for the change here indicates lower motor symptom asymmetry (Maff−Laff) at follow-up, compared with baseline. Similarly, on average, the DAT BP_ND_ asymmetry index of the putamen decreased by 7.0 ± 6.7% at follow-up. However, these changes were not yet significantly associated (Pearson’s *r* = −0.78, *P* = 0.07), likely due to the small sample size.

## Discussion

In summary, using [^18^F]FE-PE2I PET, we measured significantly lowered DAT availability in nigrostriatal regions in Parkinson’s disease compared with controls. In the striatal regions, progressive DAT losses within patients with Parkinson’s disease could be quantified at the 2-year follow-up. We also observed significant association between DAT losses in the nigrostriatal regions in Parkinson’s disease and worsening motor severity of the disease, in both cross-sectional and longitudinal studies. Lastly, we observed a significant asymmetry in DAT loss in the putamen (∼28% higher loss in the Maff hemisphere), and this was associated with the asymmetry in clinical presentation of the disease.

In cross-sectional studies, we observed ∼60% lowered DAT availability in the pre-synaptic terminals within the striatum, while it was only 30% lower in the cell bodies within the substantia nigra. This difference is consistent with previous PET studies using this tracer^6-8,10^ and post-mortem data^35^ from similar stages of the disease. The Braak model of pathology attributes most early aggregates of α-synuclein within the nigrostriatal system to the substantia nigra, from where it then spreads to the striatal areas .^36^ However, this notion has been challenged by other authors, who have suggested that the loss of pre-synaptic dopaminergic terminals in the striatum occurs first, followed by axonal and cell body losses,^37-39^ but this cannot be tested *in vivo* until PET imaging of α-synuclein pathology is possible. However, a higher loss of DAT terminals in the putamen during the initial clinical stages of the disease, as opposed to the nigra, at least supports the notion of retrograde degeneration of nigrostriatal pathway at this stage of the disease. At the same time, compensation mechanisms for DAT levels in the nigral cell bodies in early stages of the disease cannot be ruled out. A more complete understanding of the pattern of neurodegeneration in the nigrostriatal pathway would require a long-term longitudinal study in a large cohort.

Another important result was the existence of significant negative associations between MDS-UPDRS Part III scores and the DAT availability in the putamen, caudate, substantia nigra and ventral striatum. Only one previous [^18^F]FE-PE2I study has reported a significant association between motor scores (after removing tremor scores) and DAT availability in the putamen in a cohort in the medication OFF state,^10^ though other studies employing [^11^C]-PE2I have indeed reported correlations of striatal DAT binding with total UPDRS-III (OFF state) and bradykinesia rigidity scores.^40,41^ It is worth noting that our study showed a stronger association (Pearson’s *r* ∼ −0.6) between DAT availability in the putamen and total MDS-UPDRS Part III than previous studies.^10^ We primarily attribute this to a consistent medication state (OFF) between PET and clinical assessments and the slightly higher disease staging of our cohort (Hoehn–Yahr: 2) compared with previous reports (Hoehn–Yahr: 1–1.5).^10^ Additionally, we measured a significant correlation (Pearson’s *r* = −0.55) between the DAT BP_ND_ in the caudate and MDS-UPDRS Part III scores, which strengthened when tremor scores were removed (Pearson’s *r* = −0.62). This association is rarely reported in SPECT-based DAT scans, which shows a practical utility for improved resolution and sensitivity offered by PET. It should be noted, however, when using AAL template ROI for the caudate, partial volume correction was necessary to accurately quantify DAT losses in the caudate in both cross-sectional and longitudinal data.

To our knowledge, this is the first reported association between DAT availability in the substantia nigra and MDS-UPDRS Part III scores. Apart from a consistent medication state, methodological differences might have also played a role in better quantification of the nigral DAT availability in our study. First, our in-house template for the nigral ROI is based on a hand-drawn segmentations for the region from previous [^11^C]PHNO-PET studies in healthy controls, which were performed on the same scanner (HRRT) as in the present study. Second, motion blurring of images can negatively affect quantification of smaller regions, and therefore, careful motion correction based on the Polaris Vicra optical system may have mitigated these errors.

Like previous reports,^6,8,10^ the lack of correlation between nigrostriatal DAT availability and tremor scores is not surprising based on the current understanding of tremor pathophysiology. The circuit model of tremor syndromes suggests that the parkinsonian tremor is triggered by the sub-thalamic nucleus and amplified by the overactivity of the cerebello–thalamo–cortical circuit ,^42^ indicating the role of brain structures outside the nigrostriatal system in tremor generation. Removing tremor scores from MDS-UPDRS Part III strengthened the extent and magnitude of the correlations with DAT availability in all nigrostriatal regions (including caudate), which indicates this correlation is primarily driven by the part of the MDS-UPDRS III motor exam that measures bradykinesia/rigidity.

We observed significant asymmetry in DAT availability in the putamen in our cohort, with the Maff sides (contralateral to symptom onset) of the putamen and caudate showing 28 and 18% lower DAT availability, respectively. Asymmetry in the putamen was also associated with the asymmetry in the motor scores, further confirming the role of dopaminergic degeneration of the putamen in the development of motor symptoms. For most patients (all except 3 or 88%), the symptom onset side of their body had higher MDS-UPDRS scores. Similarly, most patients (all except 4 or 84%) had lower putaminal DAT BP_ND_ in the brain hemisphere contralateral to symptom onset side. We did not observe a significant asymmetry in nigral DAT availability. It is conceivable that in our cohort with average disease duration of more than 6 years, the nigral degeneration was comparable in both hemispheres (floor effect for asymmetry). Alternatively, our ROI-based analysis may not have been sensitive enough to detect the lateralization effects in a small structure such as the substantia nigra.

Results from longitudinal follow-up at the 2-year time point show 5–10% annual reduction in DAT availability in the putamen, caudate and ventral striatum, consistent with previous reports.^9,11^ The change in DAT availability in the substantia nigra was not significant, either due to floor effect for DAT loss in the cell soma or inaccurate quantification due to limited resolution. Changes in DAT availability in the putamen were strongly associated (Pearson’s *r* = 0.91) with changes in MDS-UPDRS Part III at follow-up, demonstrating the ability of [^18^F]FE-PE2I PET to track disease progression in this small cohort. At this stage of the disease, this correlation was stronger in the Maff putamen, but it is plausible that better quantification of progressive DAT losses in the Laff putamen may lead to better association with worsening motor function in advanced stages of the disease. PET measurement of asymmetry in DAT availability in the putamen and clinical asymmetry of motor symptoms were slightly reduced at the 2-year follow-up consistent with previous reports demonstrating that loss of asymmetry is the fastest predictor of disease progression.^43^ However, the changes in these measures were not significantly associated yet likely due to the small sample size (*n* = 6).

Based on the results of DAT-PET, we suspect two of the participants with Parkinson’s disease (one included in the longitudinal study) to be scans without evidence of dopamine deficit subjects due to their relatively high putamen DAT availability and unresponsiveness to carbidopa/levodopa. On average, these subjects (disease duration: 2.8 ± 2.4 years) showed lower motor severity (MDS-UPDRS Part III: 15.5 ± 2.1), with bradykinesia/rigidity score (14.0 ± 2.8) accounting for most of their disease severity. The asymmetry in their motor symptoms presentation was comparable with the average asymmetry observed in the cohort, but no asymmetry in DAT BP_ND_ was observed (DAT asymmetry index <4% in putamen and caudate). For the suspected scans without evidence of dopamine deficit subject with longitudinal data, their annual per cent reduction in DAT BP_ND_ was lower in the putamen (5% compared with 10.6 ± 2.6% for other participants) and higher in the caudate (14% compared to 9.7 ± 3.8%), with no change in the substantia nigra, though this latter value was highly heterogeneous for all participants.

Finally, we observed significantly lowered (7–12%) relative brain perfusion (*R*_1_) in the caudate, occipital and temporal lobes. These regions typically show hypometabolism in [^18^F]FDG PET studies in Parkinson’s disease.^44,45^ Moreover, we previously demonstrated with [^11^C]UCB-J PET in 30 subjects with Parkinson’s disease and 30 controls (most patients with Parkinson’s disease from this study’s cross-sectional cohort were included in that study) similar reductions in [^11^C]UCB-J *R*_1_ in the caudate and occipital lobes.^46^ Indeed, previous studies have validated the use of [^18^F]FE-PE2I *R*_1_ against relative cerebral blood flow measure from [^15^O]H_2_O PET in individuals with Parkinson’s disease and healthy individuals.^32^ However, beyond these group differences, we did not observe any other significant clinical correlate with *R*_1_.

Our study was similar in design when compared with recent cross-sectional^10^ and longitudinal^11^ PET investigations with [^18^F]FE-PE2I, but with some differences in patient demographics. In particular, our cross-sectional cohort was composed of patients who were slightly more advanced in disease staging and motor symptoms (Hoehn–Yahr: 2, MDS-UPDRS III: 30 ± 12, disease duration: 6.1 ± 4.5 years at age: 64.4 ± 6.7 years) than the cross-sectional cohort (medication off state) reported by Kerstens *et al*.^10^ (Hoehn–Yahr: 1, MDS-UPDRS III: 22 ± 10, disease duration: 3 years at age: 68 years). The longitudinal cohort in this study was more similar to the (medication off) cohort reported in the study by Kerstens *et al*.^11^ in terms of age, disease duration and MDS-UPDRS III at baseline but still had differences in disease staging (Hoehn–Yahr 2 versus 1). Future [^18^F]FE-PE2I PET studies should focus on the prodromal (rapid eye movement sleep behaviour disorder and other non-motor presentation) stage of the disease, as well as on the trajectory of DAT loss from longer follow-up of patients in more advanced stages of Parkinson’s disease.

## Conclusion

We validated the clinical use of [^18^F]FE-PE2I for DAT-PET imaging in independent cross-sectional and longitudinal cohorts of individuals with mild and bilateral Parkinson’s disease. Our results show that [^18^F]FE-PE2I PET is indeed a sensitive biomarker of motor severity in Parkinson’s disease. DAT availability in all nigrostriatal regions (including substantia nigra) was significantly associated with total MDS-UPDRS Part III, but not tremor. This association is primarily driven by the parts of the motor exam that measure bradykinesia and rigidity. Asymmetry in motor symptoms could be explained based on asymmetric losses of dopaminergic terminals within the putamen. In addition, [^18^F]FE-PE2I PET was sensitive to intra-individual changes in DAT availability in most regions except the substantia nigra. Change in DAT availability in the putamen was associated with change in motor burden of the disease, though this requires further validation in a larger cohort. In summation, not only would [^18^F]FE-PE2I PET be a promising imaging marker for future clinical trials with novel interventions for Parkinson’s disease but also an excellent candidate for translation to routine clinical use.

## Supplementary Material

fcae345_Supplementary_Data

## Data Availability

The data and code that support the findings of this study are available from the corresponding author, upon reasonable request.
