# Supplementary material for: Clinical correlates of dopamine transporter availability in cross-sectional and longitudinal studies with [18F]FE-PE2I PET: independent validation with new insights
Source: Brain Commun. 2024 Oct 2;6(5):fcae345. doi: 10.1093/braincomms/fcae345 (PMC11487911; doi:10.1093/braincomms/fcae345)
Supplement: fcae345_Supplementary_Data [file fcae345_supplementary_data.pdf]

Supporting Information

Validation of SRTM2 against SRTM for [^{18}F]FE-PE2I

While previous studies have validated the use of SRTM (with cerebellum as reference region) for [^{18}F]FE-PE2I quantification [1, 2], SRTM fits to time activity curves often provide noisy results in regions of low binding ($BP_{\mathrm{ND}} \sim 0$, extra-striatal regions for [^{18}F]FE-PE2I), particularly noticeable in parametric images (Supplementary Figure 1, top row). Often, SRTM2 (with a population-averaged k_2 parameter for the reference region) provides a lower-noise alternative to the SRTM [3], thus improving the PET quantification (Supplementary Figure 1, bottom row). Since SRTM2 has not been previously used to model [^{18}F]FE-PE2I's kinetics, we validated the SRTM2 fit (with population-averaged k_2 parameter, 0.096 min^{-1}, for the cerebellum) against the SRTM. A high degree of correlation was observed between SRTM2 and SRTM PET outcome measures in controls (Pearson's correlation coefficient for BP_{ND}: 0.997, for R_1: 0.983) and PD patients (Pearson's correlation coefficient for BP_{ND}: 0.999, for R_1: 0.979) in the nigrostriatal regions (Supplementary Figure 2). Further, Bland-Altman plots showed only a minor difference between the outcomes in both populations (Supplementary Figure 2). Therefore, all analysis and results reported in this paper used BP_{ND} and R_1 outcomes from SRTM2 fit to the PET data.

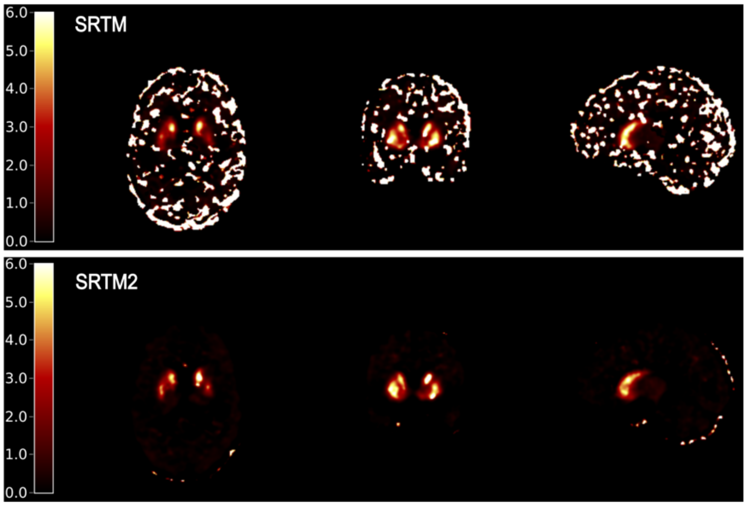

Supplementary Figure 1: Representative example of BP_{ND} images from SRTM and SRTM2 fits (ref = Cerebellum) to PET data. SRTM2 fit shows lower noise in the extra-striatal regions. Abbreviations- BP_{ND}: Binding Potential (relative to non-displaceable fraction), SRTM: Simplified Reference Tissue Model.

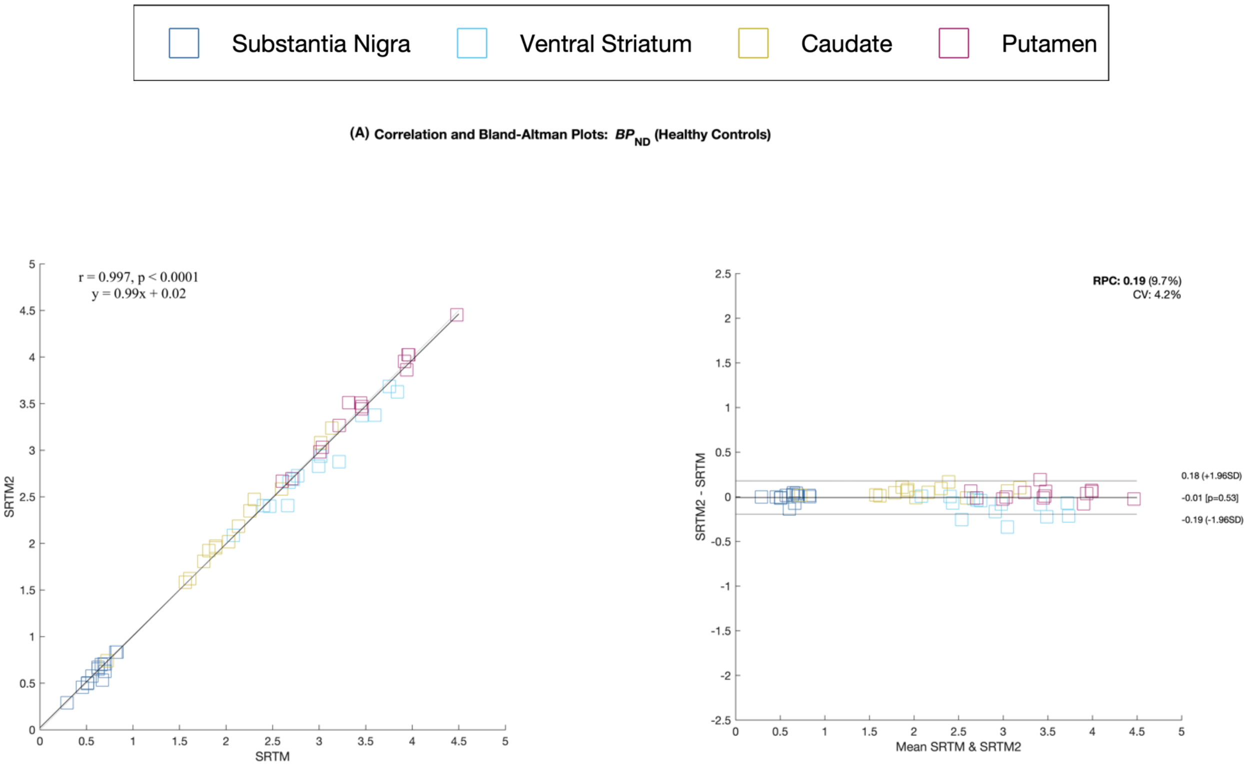

Supplementary Figure 2-A: Pearson's correlation coefficient ($r = 0.997$, $p < 0.0001$, $n = 56$ [14 subjects x 4 regions]) and Bland-Altman plots for BP_{ND} in healthy control subjects using SRTM and SRTM2. CV = Coefficient of Variation, RPC = Reproducibility Coefficient, BP_{ND} = Binding Potential (relative to non-displaceable fraction), SRTM = Simplified Reference Tissue Model

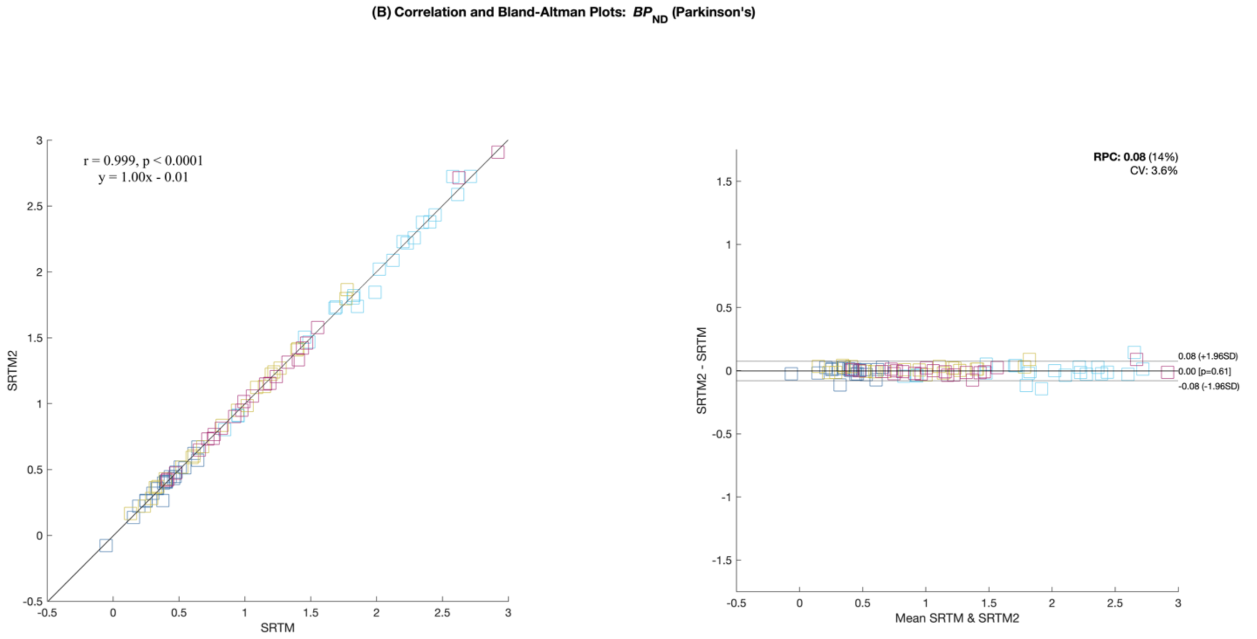

Supplementary Figure 2-B: Pearson's correlation coefficient ($r = 0.999$, $p < 0.0001$, $n = 80$ [20 subjects x 4 regions]) and Bland-Altman plots for BP_{ND} in Parkinson's subjects using SRTM and SRTM2. CV = Coefficient of Variation, RPC = Reproducibility Coefficient, BP_{ND} = Binding Potential (relative to non-displaceable fraction), SRTM = Simplified Reference Tissue Model

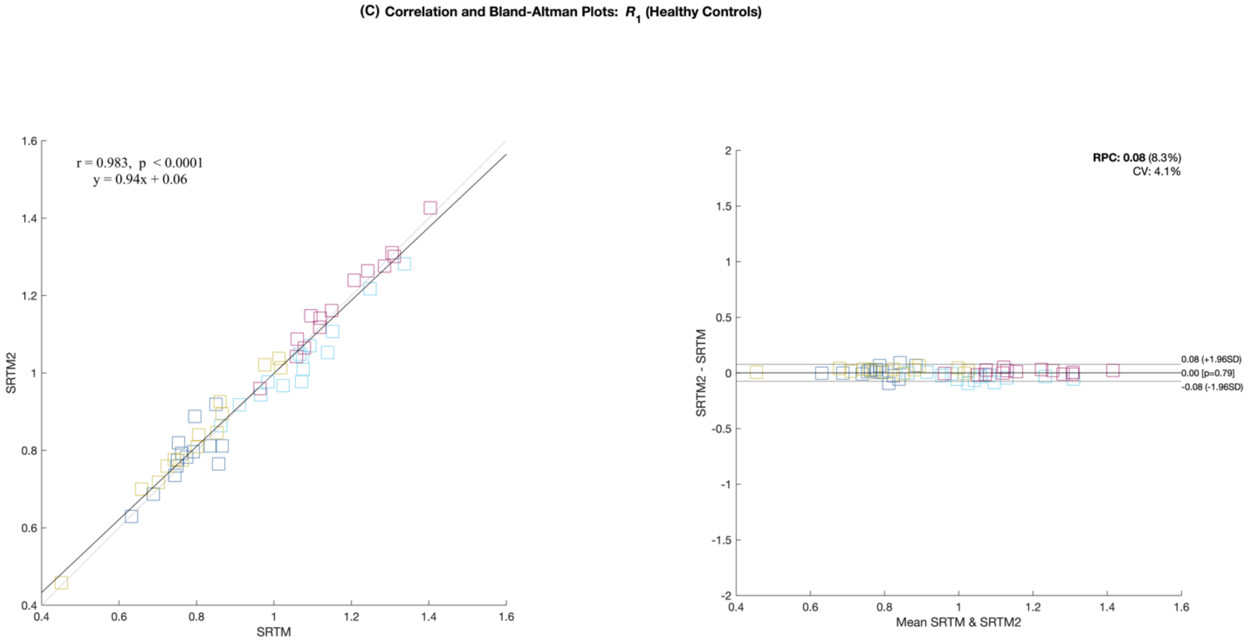

Supplementary Figure 2-C: Pearson's correlation coefficient (r = 0.983 p < 0.0001, n = 56 [14 subjects x 4 regions]) and Bland-Altman plots for R_1 in healthy control subjects using SRTM and SRTM2. CV = Coefficient of Variation, RPC = Reproducibility Coefficient, R_1 = Relative Tracer Uptake, SRTM = Simplified Reference Tissue Model

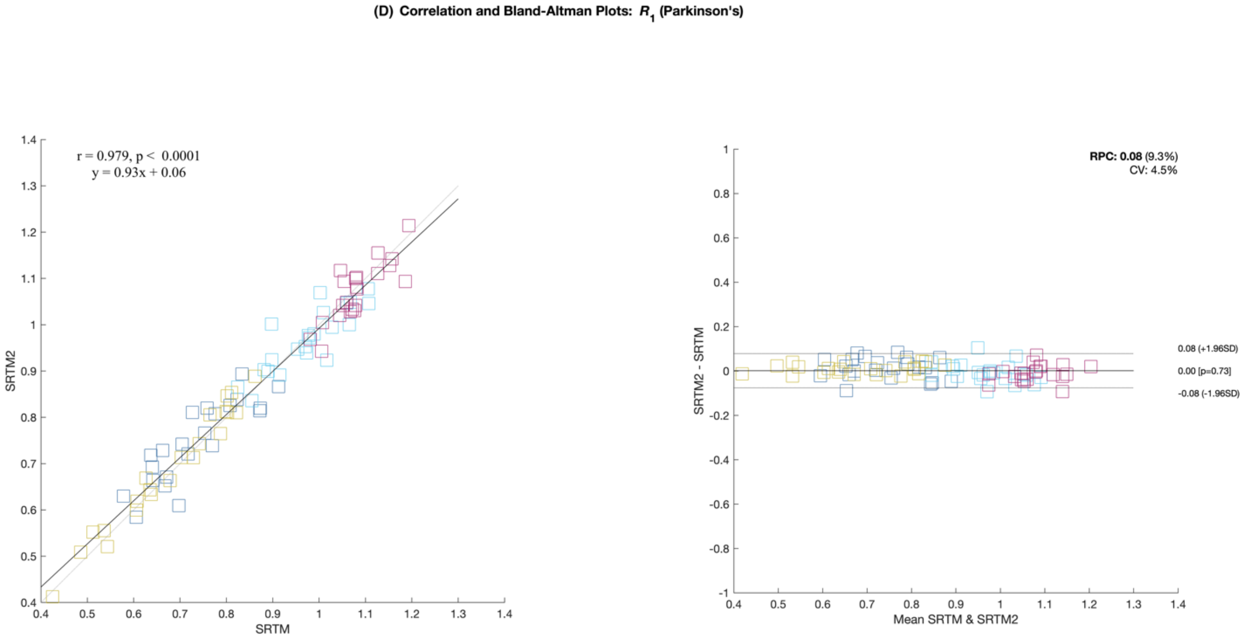

Supplementary Figure 2-D: Pearson's correlation coefficient (r = 0.979, p < 0.0001, n = 80 [20 subjects x 4 regions]) and Bland-Altman plots for R_1 in Parkinson's subjects using SRTM and SRTM2. CV = Coefficient of Variation, RPC = Reproducibility Coefficient, R_1 = Relative Tracer Uptake, SRTM = Simplified Reference Tissue Model

Radiosynthesis and quality-control details for [^{18}F]FE-PE2I production

Radiosynthesis of [^{18}F]FE-PE2I is depicted in Scheme 1. It was produced as described previously [4] via nucleophilic radiofluorination of the tosylate precursor in the presence of tetraethylammonium bicarbonate (TEAB) as phase transfer catalysts instead of Kryptofix 222.

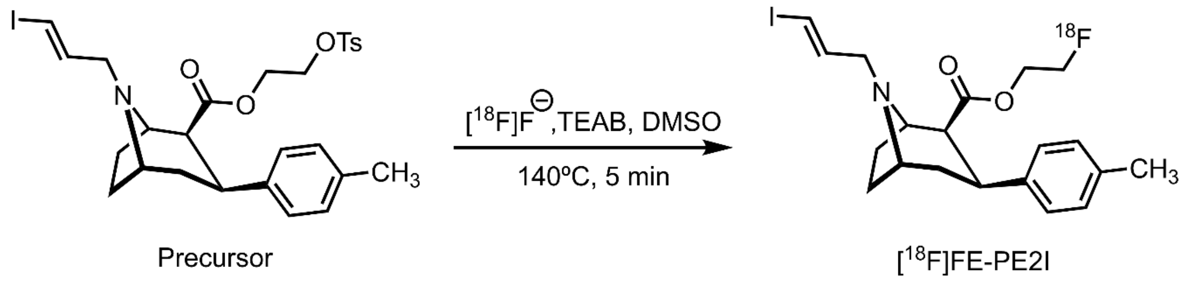

Scheme 1. Radiosynthesis of [^{18}F]FE-PE2I.

The requisite aqueous [^{18}F]fluoride was produced via the ^{18}O(p,n)^{18}F nuclear reaction. This nuclear reaction is brought about by bombarding a high yield niobium [^{18}O]water target assembly with a proton source using the 16.4 MeV GEMS PET trace cyclotron (GE, Uppsala, Sweden). The cyclotron produced [^{18}F]fluoride solution was transferred to a GE TracerLab FXN or FXN-Pro in a lead-shielded hot cell, where the radioactivity is trapped on an ion-exchange ^{18}F separation cartridge. Then the [^{18}F]fluoride was eluted off the cartridge into the reaction vessel with a solution of tetraethylammonium bicarbonate (TEAB, 2 mg) in 1 mL of acetonitrile/water solution (7:3, v/v). The [^{18}F]fluoride solution was then dried at 70ºC for 5 min by azeotropic distillation, followed by the addition of 2x1 mL acetonitrile and azeotropic distillation for 3 min each, and heating at 100ºC in vacuo for 4 min. After cooling to 60 ºC, a solution of the tosylate precursor (0.4-1 mg) in DMSO (0.5 mL) was added to the reaction vessel, and the resulting solution was heated at 140 ºC for 5 min. After cooling to 50 ºC, the reaction mixture was diluted with 10 mL de-ionized (DI) water and passed through a Waters Classic C18 SepPak cartridge to remove unreacted [^{18}F]fluoride. The trapped crude product was then eluted off the SepPak with 2.5 mL acetonitrile, diluted with 2.5 mL DI water and then loaded onto the semi-preparative HPLC column for purification.

The semi-preparative HPLC system uses a semi-preparative column (Gemini C18, 5 µm, 10 × 250 mm) eluted under isocratic conditions with a mixture of 70% acetonitrile and 30% 0.1 M aqueous ammonium carbonate solution$_{(v/v)}$ at a flow rate of 4 mL/min. The eluent is monitored by a UV detector and a radioactivity detector. The fraction containing [^{18}F]FE-PE2I was collected, diluted with a solution of 400 mg ascorbic acid (USP grade) in 50 mL of DI water, and passed through a Waters Light C18 SepPak cartridge. The cartridge was rinsed with a solution of 10 mg ascorbic acid (USP) in 10 mL of 10% aq. ethanol and dried by inert gas. The radioactive product trapped on the SepPak was recovered by eluting with 1 mL of absolute ethanol (USP) followed by a solution of 3 mg ascorbic acid (USP) in 3 mL of saline (USP) and collected into the module product vial precharged with a solution of 7 mg ascorbic acid (USP) in 7 mL saline (USP) and 100 µL of 8.4% (or 200 µL of 4.2%) sodium bicarbonate (USP). Finally the PET

drug solution was passed through a sterile 0.22 μm membrane filter (33 mm Millex-GV) for terminal sterilization and collected in a sterile, pyrogen-free collection vial affording a formulated solution of [18F]FE-PE2I ready for dispensing and injection.

Other Supplemental Information

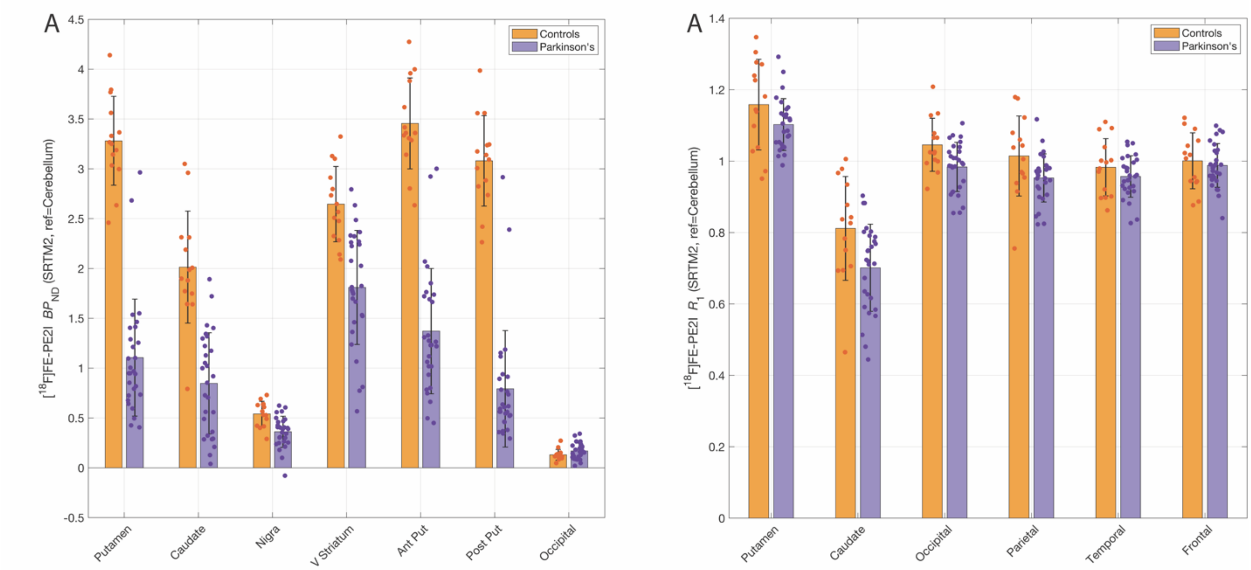

Supplementary Figure 3: Group comparison of dopamine transporter availability (A) and relative cerebral blood flow (B) between Parkinson's disease (n=28) and healthy control (n=14) subjects without partial volume correction (PVC). Generally, the group differences are very similar to Figure 1 (partial volume corrected) with the exception of R_1 in parietal lobe. Parietal lobe R_1 is 7% lowered in PD without PVC, but 9% higher in PD with PVC. Abbreviations - BP_{ND}: Binding Potential (relative to non-displaceable fraction), SRTM2: Simplified Reference Tissue Model 2, ref: reference region, V Striatum: Ventral Striatum, Ant Put: Anterior Putamen, Post Put: Posterior Putamen.

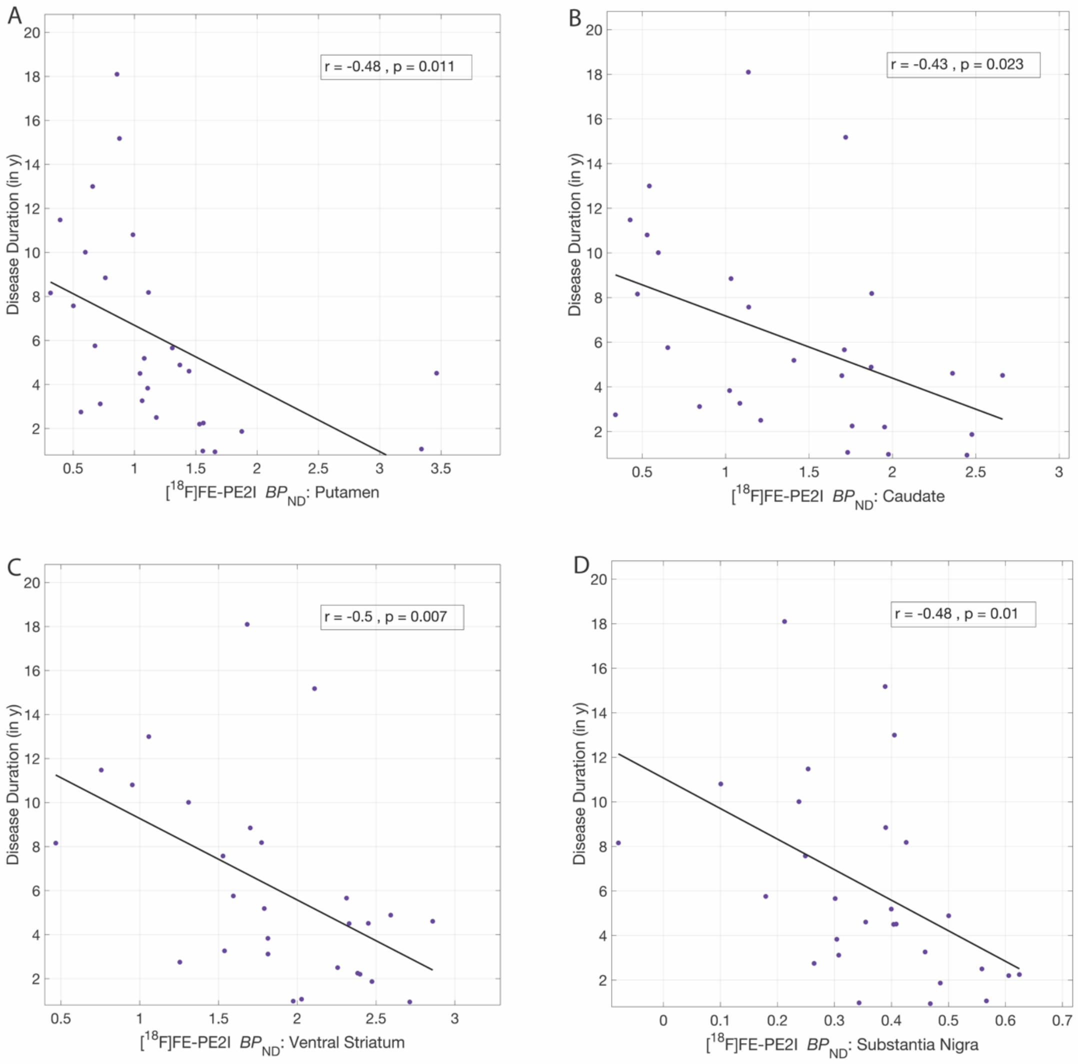

Supplementary Figure 4: Association (Pearson's, n=28) of disease duration (from self-reported symptom onset in years) with dopamine transporter availability in the putamen (A), caudate (B), ventral striatum (C) and substantia nigra (D). Note that the correlation with the putamen BP_{ND} would be stronger (r= -0.57, p=0.002) if the two Parkinson's subjects without evidence of dopamine deficit are removed from this analysis. Abbreviations – BP_{ND}: Binding Potential (relative to non-displaceable fraction).

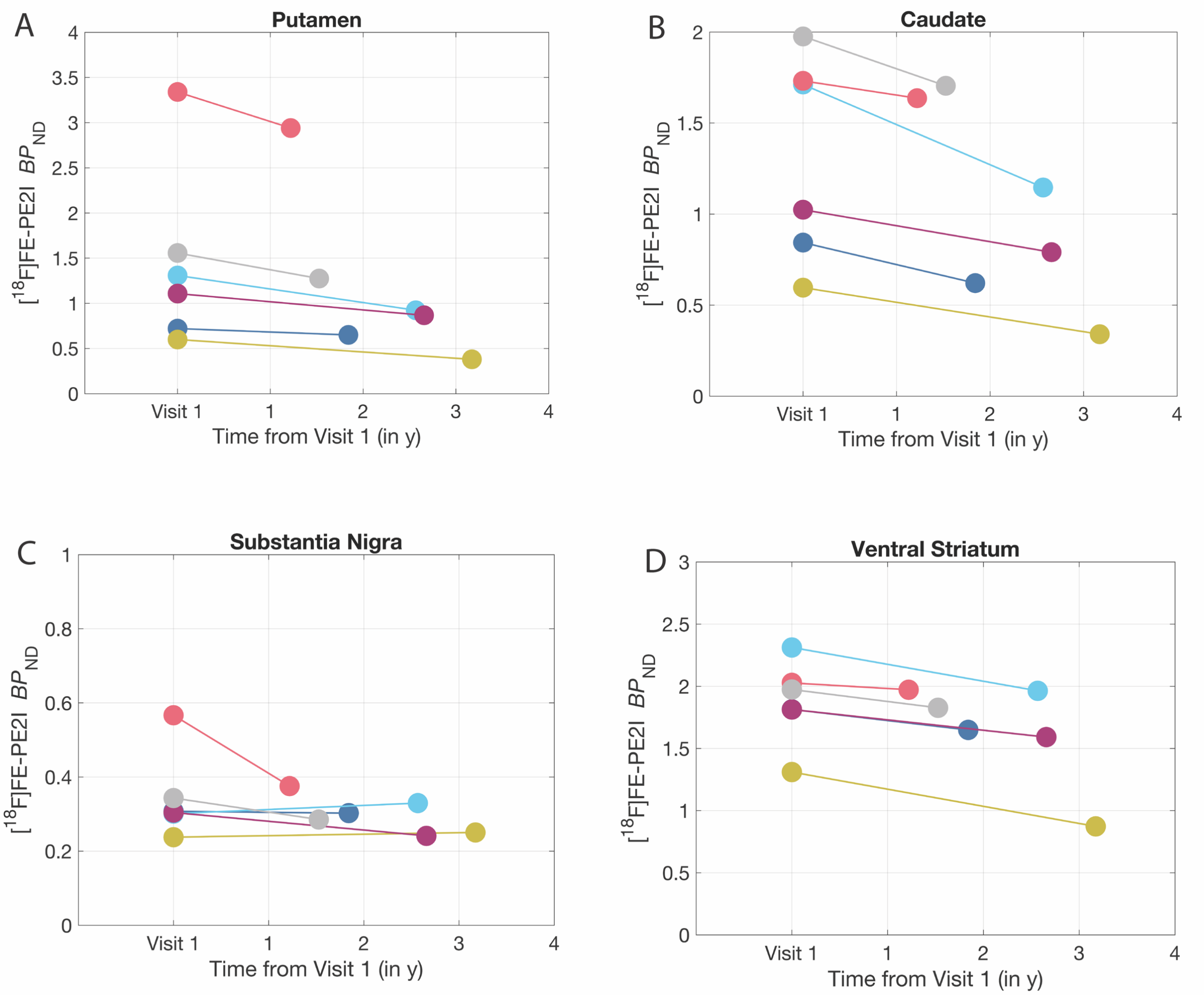

Supplementary Figure 5: Trajectories of dopamine transporter availability ([^{18}F]FE-PE2I BP_{ND}) for 6 individuals with Parkinson's disease in the longitudinal study. Abbreviations – BP_{ND}: Binding Potential (relative to non-displaceable fraction).

Explanation regarding a subject with negative BP_{ND} value in the substantia nigra

In Figure 2D of the main manuscript, a Parkinson's disease patient is shown to a negative value of BP_{ND} in the substantia nigra. This value is explained by modeling choices, specifically using the SRTM2 with *a* fixed population-averaged value of k_2' for the reference region.

For example, for the same subject if we instead used the Wu and Carson [3] implementation of SRTM2 – where each scan's fixed k_2' value is chosen as the median from voxel-wise SRTM fits– the nigral BP_{ND} for this subject would become greater than 0 (0.12), and that would still be the lowest BP_{ND} value of all subjects. To instill further confidence, we present below the correlations between MDS-UPDRS part III scores and nigral DAT BP_{ND} using (1) SRTM2 (Wu and Carson) and (2) SRTM2 with population-averaged k_2'. The correlation coefficients (-0.47 and -0.46, respectively) and statistical significance levels (p=0.015 and p=0.018, respectively) are similar in each case (see Figure S5).

The reason for the slightly negative value of BP_{ND} may be attributed to this subject's k_2' value (estimated as 0.03 min^{-1} from Wu and Carson implementation) being uncharacteristically lower than the population-

averaged k_2' value (0.09 min^{-1}). Figure S6 shows nigral SRTM2 BP_{ND} (ref=Cerebellum, fixed k_2') computed from regional time activity curves for this subject as a function of the fixed k_2' value – note that even for a large variation in k_2', the change in BP_{ND} values is small (between -0.1 and 0.1). Therefore, even though SRTM2 with a population-averaged k_2' may cause an underestimation of BP_{ND} values in regions with low DAT and in rare cases a negative BP_{ND} value, we still believe that overall this is an excellent modeling approach for this tracer in most situations due to (1) its simplicity, and (2) excellent agreement across subjects and ROIs with the SRTM (see, Validation of SRTM2 against SRTM for [^{18}F]FE-PE2I section in this file).

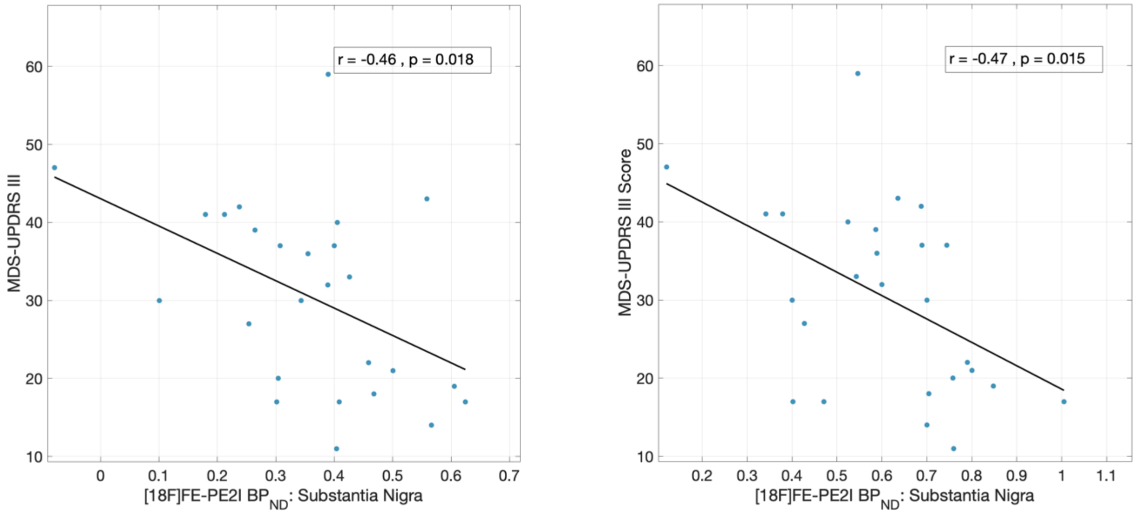

SRTM2 with fixed k_2' SRTM2 (Wu and Carson[3])

Supplementary Figure 6: SRTM2 with fixed k_2' (left) and the Wu and Carson implementation (right). The Pearson's correlation coefficient of MDS-UPDRS with nigral dopamine transporter density are similar for the fixed k_2' (r = -0.46, p = 0.018, n = 26) and Wu and Carson (r = -0.47, p = 0.015, n = 26) implementation. Abbreviations - BP_{ND}: Binding Potential (relative to non-displaceable fraction), SRTM2: Simplified Reference Tissue Model, MDS-UPDRS: Movement Disorders Society – Unified Parkinson's Disease Rating Scale.

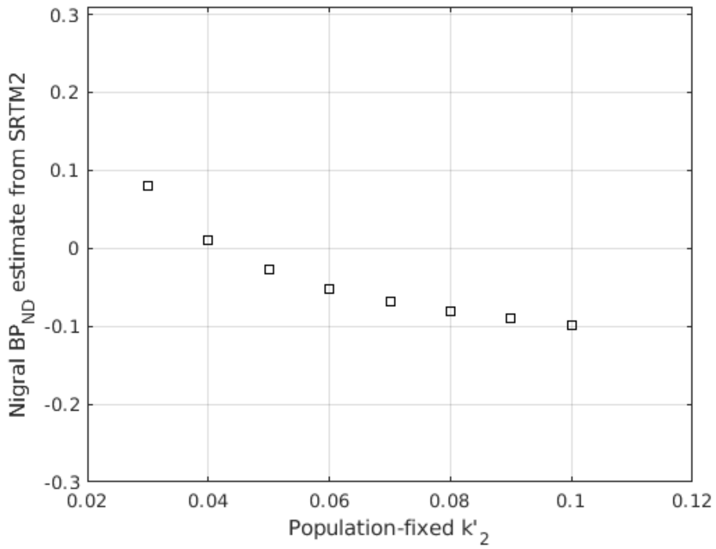

Supplementary Figure 7: Data points show nigral BP_{ND} estimate (y-axis) for a range of population-averaged k_2' values (x-axis), a fixed-parameter in SRTM2 fits to regional time-activity curves, for the subject with negative nigral BP_{ND} value reported in the main analysis. The variation in the

BP_{ND} estimates is small and the BP_{ND} value can be negative if the population-averaged k_2' is higher than subject's k_2'. Abbreviations - BP_{ND}: Binding Potential (relative to non-displaceable fraction), SRTM2: Simplified Reference Tissue Model.

Supplementary References

1. Delva, A., et al., *Quantification and discriminative power of (18)F-FE-PE2I PET in patients with Parkinson's disease.* Eur J Nucl Med Mol Imaging, 2020. **47**(8): p. 1913-1926.
2. Sasaki, T., et al., *Quantification of dopamine transporter in human brain using PET with 18F-FE-PE2I.* J Nucl Med, 2012. **53**(7): p. 1065-73.
3. Wu, Y. and R.E. Carson, *Noise reduction in the simplified reference tissue model for neuroreceptor functional imaging.* J Cereb Blood Flow Metab, 2002. **22**(12): p. 1440-52.
4. Stepanov, V.K., R; Raus, L; Loog, O; Hiltunen, J; Halldin, C, *An efficient one-step radiosynthesis of [18F]FE-PE2I, a PET radioligand for imaging of dopamine transporters.* J Label Compd Radiopharm, 2012. **55**(6): p. 206-210.
